# High-quality chromosome-scale genomes facilitate effective identification of large structural variations in hot and sweet peppers

**DOI:** 10.1093/hr/uhac210

**Published:** 2022-09-19

**Authors:** Joung-Ho Lee, Jelli Venkatesh, Jinkwan Jo, Siyoung Jang, Geon Woo Kim, Jung-Min Kim, Koeun Han, Nayoung Ro, Hea-Young Lee, Jin-Kyung Kwon, Yong-Min Kim, Tae-Ho Lee, Doil Choi, Allen Van Deynze, Theresa Hill, Nir Kfir, Aviad Freiman, Nelson H Davila Olivas, Yonatan Elkind, Ilan Paran, Byoung-Cheorl Kang

**Affiliations:** Department of Agriculture, Forestry and Bioresources, Research Institute of Agriculture and Life Sciences, Plant Genomics Breeding Institute, College of Agriculture and Life Sciences, Seoul National University, Seoul 08826, Republic of Korea; Department of Agriculture, Forestry and Bioresources, Research Institute of Agriculture and Life Sciences, Plant Genomics Breeding Institute, College of Agriculture and Life Sciences, Seoul National University, Seoul 08826, Republic of Korea; Department of Agriculture, Forestry and Bioresources, Research Institute of Agriculture and Life Sciences, Plant Genomics Breeding Institute, College of Agriculture and Life Sciences, Seoul National University, Seoul 08826, Republic of Korea; Department of Agriculture, Forestry and Bioresources, Research Institute of Agriculture and Life Sciences, Plant Genomics Breeding Institute, College of Agriculture and Life Sciences, Seoul National University, Seoul 08826, Republic of Korea; Department of Agriculture, Forestry and Bioresources, Research Institute of Agriculture and Life Sciences, Plant Genomics Breeding Institute, College of Agriculture and Life Sciences, Seoul National University, Seoul 08826, Republic of Korea; Department of Agriculture, Forestry and Bioresources, Research Institute of Agriculture and Life Sciences, Plant Genomics Breeding Institute, College of Agriculture and Life Sciences, Seoul National University, Seoul 08826, Republic of Korea; Vegetable Research Division, National Institute of Horticultural and Herbal Science, Rural Development Administration, Jeonju 55365, Republic of Korea; National Agrobiodiversity Center, National Institute of Agricultural Sciences, Rural Development Administration, Jeonju 54874, Republic of Korea; Department of Agriculture, Forestry and Bioresources, Research Institute of Agriculture and Life Sciences, Plant Genomics Breeding Institute, College of Agriculture and Life Sciences, Seoul National University, Seoul 08826, Republic of Korea; Department of Agriculture, Forestry and Bioresources, Research Institute of Agriculture and Life Sciences, Plant Genomics Breeding Institute, College of Agriculture and Life Sciences, Seoul National University, Seoul 08826, Republic of Korea; Korean Bioinformation Center, Korea Research Institute of Bioscience and Biotechnology, Daejeon 34141, Republic of Korea; Genomics Division, National Institute of Agricultural Sciences, Rural Development Administration, Jeonju 54874, Republic of Korea; Department of Agriculture, Forestry and Bioresources, Research Institute of Agriculture and Life Sciences, Plant Genomics Breeding Institute, College of Agriculture and Life Sciences, Seoul National University, Seoul 08826, Republic of Korea; Department of Plant Sciences, University of California, Davis, CA 95616, USA; Department of Plant Sciences, University of California, Davis, CA 95616, USA; NRGene, 5 Golda Meir St., Ness Ziona 7403649, Israel; Top Seeds International Ltd. Moshav Sharona, 1523200, Israel; BASF’s vegetable seeds business. Napoleonsweg 152 6083 AB Nunhem, the Netherlands; Pilpel Seeds Ltd. Nes Ziona, 7414001, Israel; Institute of Plant Science, Agricultural Research Organization, The Volcani Center, Rishon LeZion, Israel; Department of Agriculture, Forestry and Bioresources, Research Institute of Agriculture and Life Sciences, Plant Genomics Breeding Institute, College of Agriculture and Life Sciences, Seoul National University, Seoul 08826, Republic of Korea

## Abstract

Pepper (*Capsicum annuum*) is an important vegetable crop that has been subjected to intensive breeding, resulting in limited genetic diversity, especially for sweet peppers. Previous studies have reported pepper draft genome assemblies using short read sequencing, but their capture of the extent of large structural variants (SVs), such as presence–absence variants (PAVs), inversions, and copy-number variants (CNVs) in the complex pepper genome falls short. In this study, we sequenced the genomes of representative sweet and hot pepper accessions by long-read and/or linked-read methods and advanced scaffolding technologies. First, we developed a high-quality reference genome for the sweet pepper cultivar ‘Dempsey’ and then used the reference genome to identify SVs in 11 other pepper accessions and constructed a graph-based pan-genome for pepper. We annotated an average of 42 972 gene families in each pepper accession, defining a set of 19 662 core and 23 115 non-core gene families. The new pepper pan-genome includes informative variants, 222 159 PAVs, 12 322 CNVs, and 16 032 inversions. Pan-genome analysis revealed PAVs associated with important agricultural traits, including potyvirus resistance, fruit color, pungency, and pepper fruit orientation. Comparatively, a large number of genes are affected by PAVs, which is positively correlated with the high frequency of transposable elements (TEs), indicating TEs play a key role in shaping the genomic landscape of peppers. The datasets presented herein provide a powerful new genomic resource for genetic analysis and genome-assisted breeding for pepper improvement.

## Introduction

The availability of reference genomes for many crops has generated a powerful platform for discovering genetic variation and identifying marker–trait associations for agriculturally important traits. Until recently, small variations between plant genomes were identified by comparing a single genome typically not of high quality but rather a single pass with short reads to a high-quality reference genome [1,2]. This approach has supported the efficient discovery of small insertion/deletion (InDel) variants and single-nucleotide polymorphisms (SNPs) between the two genomes of interest but has been less useful, or even failed, in identifying large structural variants (SVs) when they are either missing or highly diverged from the reference genome. Furthermore, a single genome cannot encompass the full complement of the genetic diversity of a species [3, 4]. SVs, such as presence–absence variation (PAV) and copy-number variation (CNV), cover large genomic regions from 1 kb to several megabases and can functionally influence the genome to a greater extent than SNPs or small InDels [3–6]. However, capturing the full spectrum of all genomic variants in plant species with complex genomes remains challenging.

The concept of “pan-genome” was introduced to capture the full spectrum of genome variants between individuals of a species [7]. Broadly speaking, a pan-genome represents the collective genomic information of a species; it is generally grouped into core genes shared across all or most individuals and variable or accessory genes that are found in only a subset of individuals [7–10]. An increasing number of pan-genomes have been assembled, including for soybean [11], cabbage [9], tomato [12], rapeseed [13], maize [14], purple false brome [15], rice [16–18], wheat [19], and barley [6]. Several crop pan-genomes exhibit large SVs, such as PAVs or CNVs, in key genes associated with important agricultural traits [6, 13, 20].

Pepper (*Capsicum* spp.) is one of the earliest domesticated crops in the Solanaceae family and is believed to have originated in Central and South America [21]. Pepper accessions can be broadly classified as either “hot” or “sweet” according to the pungency of their fruits. As a result of domestication, breeding, and market demand, hot peppers usually bear small fruits, whereas sweet peppers tend to have larger fruits. *Capsicum* species display extensive variation in agriculturally important traits, such as plant architecture, fruit color and morphology, accumulation of secondary metabolites, yield, and response to a number of biotic and abiotic stresses [22, 23]. Efficient utilization of pepper genetic resources is essential to help face challenges arising from adverse climatic change and rapidly evolving pathogens and, ultimately, to meet the current and future global demand for pepper fruits [24].

Following the release of the first *C. annuum* reference genome in 2014 [25, 26], multiple pepper genome assemblies have been released using short read sequencing approaches [27–29] and have opened the door to better understanding pepper genome organization and to accelerating breeding. In addition, these initial genome sequences have led to the identification of specific genomic regions subjected to domestication and improvement [24, 30–32]. An earlier version of a pepper pan-genome using *C. annuum* “Zunla” as a reference genome facilitated genome-wide association studies (GWAS) for carotenoid contents in *Capsicum* species [33]. The complement of genomic variation was identified in these studies based on pepper genome assemblies developed from short reads, which have inherent limitations, such as low continuity and low coverage of genetic variants [25, 26, 29], as well as poor resolution, especially in low-recombining regions [28]. Overall, the current state of pepper genome assemblies has limited applications for the capture of large genome SVs, including CNVs and PAVs, by short read–based genome sequencing and reference-based approaches. Therefore, a pan-genome developed using high-quality reference genomes is an important and necessary step toward a comprehensive survey of domestication, improved utilization of genomic and genetic resources, and a more inclusive characterization of pepper gene functions and breeding potential.

In this study, we assembled a chromosome-scale reference genome of the *C. annuum* sweet pepper-type cultivar ‘Dempsey’ using a combination of long-read and short-read sequencing, complemented with high-resolution chromosome conformation capture (Hi-C) scaffolding and optical mapping technologies, with the aim to improve the overall assembly quality. We exploited the resulting high-quality ‘Dempsey’ reference genome for the detection of novel loci associated with fruit orientation. We also sequenced the genomes of important pepper accessions including sweet and hot peppers by long-read and/or linked-read methods. We combined all genome assemblies into a pepper pan-genome using ‘Dempsey’ as the reference, or pivot genome, followed by the identification of large SVs. Finally, we validated SVs associated with important agricultural traits using this pepper pan-genome.

## Results

### *De novo* genome assembly of the ‘Dempsey’ reference genome

**Figure 1:**
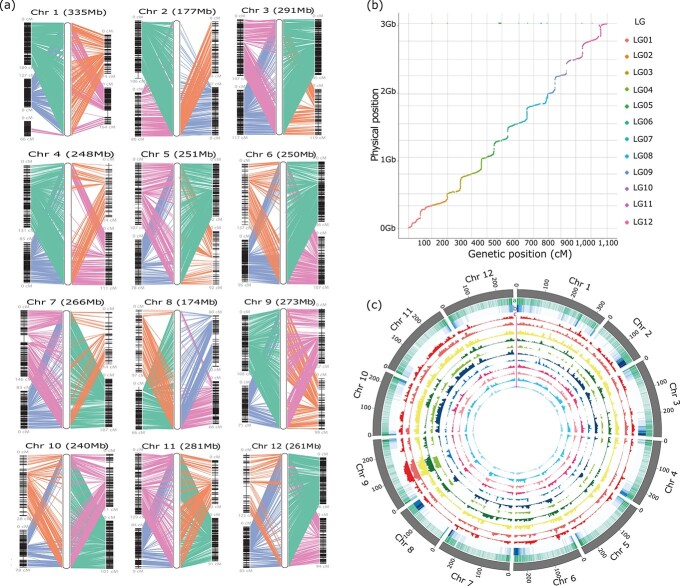
Genomic landscape of the pivot genome ‘Dempsey’ and the pseudomolecules of 11 other pepper accessions. (a) Reconstructed chromosomes for the genome of the *Capsicum annuum* cultivar ‘Dempsey’ (v1.0). Four different pepper genetic maps, FA3200929 (green), NM.200929 (orange), PD.1st.200929 (blue), and array.200929 (pink), were combined with different weights to reconstruct all ‘Dempsey’ chromosomes. Left, side-by-side alignments between ‘Dempsey’ chromosomes (chr, shown in white) and the linkage groups (two shown on each side of each ‘Dempsey’ chr); color-coded connecting lines indicate marker locations. (b) Marey map representation of ‘Dempsey’ chromosomes. The cumulative physical length of the chromosomes is shown on the y-axis. The genetic distances derived from the ‘Perennial’ × ‘Dempsey’ cross are given along the x-axis. A total of 84 SNPs and 1911 bins and including 23 scaffolds were used to construct the ‘Perennial’ × ‘Dempsey’ genetic map. (c) Circular map of presence–absence variants (PAVs) in 11 pepper genomes against the pivot genome ‘Dempsey’. Track a, gene density; Track b, density of transposable elements calculated in 200-kb windows; Tracks in c, PAVs identified in 200-kb windows; the tracks (from outside to inside) indicate ‘CC090’ (dark red), ‘CC260’ (light red), ‘ThaiHot’ (yellow), ‘MR’ (dark green), ‘Perennial’ (light green), ‘CV3’ (dark blue), ‘UCD10X’ (light blue), ‘LaMuyo-01’ (dark pink), ‘I19–702-1’ (light pink), ‘PG1’ (dark cyan), and ‘Maor’ (light cyan).

We sequenced and assembled a high-quality genome for the *C. annuum* cultivar ‘Dempsey’ by integrating Pacific Biosciences (Pacbio, Menlo Park, USA) long reads, Illumina short reads (Illumina Inc., Carlsbad, USA), optical mapping, and Hi-C (Table S1). We generated approximately 279 Gb (~90× coverage) of raw long-read data using the PacBio Sequel platform. The assembled contigs after error correction covered 3.03 Gb of sequence, with an N50 (the size of the shortest contig fragment from 50% of the total genome) value of 18.3 Mb. We then used 815 million Hi-C sequencing reads to align the assembled PacBio contigs above, which increased the N50 value to 257.6 Mb (Table S1, S2). Optical mapping generated 157× genome coverage, with an N50 value of 236.7 kb (Table S3). The integration of all scaffolds obtained from PacBio, Hi-C, and optical mapping data covered 3.05 Gb of genome sequence, with a final scaffold N50 value of 260.6 Mb, which is close to chromosome size (Table S1). For any incongruences between the Hi-C and optical maps, we manually adjusted BioNano super-scaffolds with previously published pepper genetic maps (Fig. 1a) and ‘Perennial’ × ‘Dempsey’ genetic map [34, 35] (Fig. 1b). Pearson’s correlation coefficients between the ‘Dempsey’ genome assembly and the genetic maps ranged from 0.928 to 1 for all chromosomes (Supplementary Fig. S1).

The final ‘Dempsey’ genome assembly consisted of 3.03 Gb of sequence arranged into 141 scaffolds, with a scaffold N50 value of 260.6 Mb. We then assembled them into 15 super-scaffolds and 18 scaffolds representing the 12 ‘Dempsey’ chromosomes (Fig. 1a; Table S4; Supplementary Dataset S1), with much improved genome quality (smaller gap sizes and considerably fewer gaps) than previous pepper genomes (Table S5).

### Use of ‘Dempsey’ reference genome for GWAS of fruit orientation

**Figure 2:**
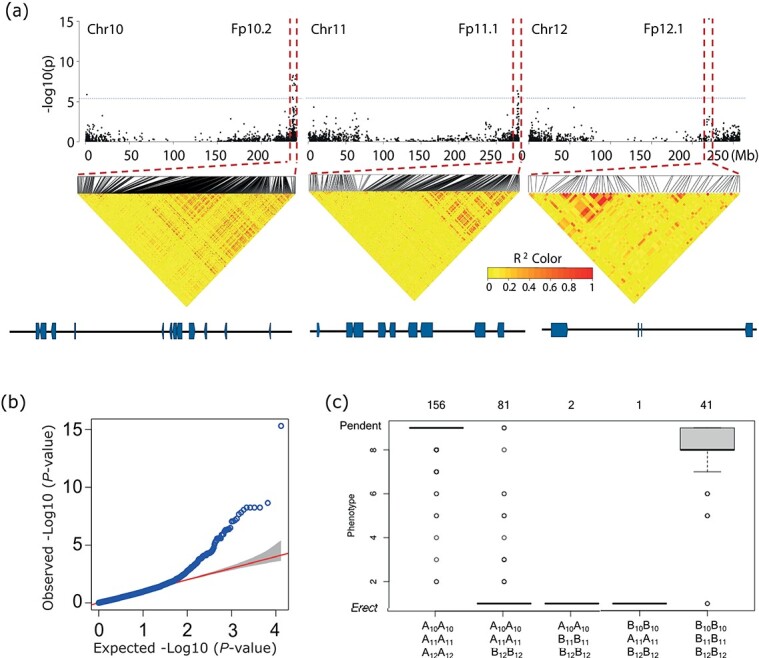
Genome-wide association study (GWAS) for pepper fruit orientation in the 288 *C. annuum* association panel. (a) Manhattan plots of chromosome (Chr) 10, Chr 11, and Chr 12 showing significant marker–trait associations with –Log_10_*P* > 6.27 (represented by a blue horizontal line) and a false discovery rate < 0.05 for fruit orientation. Dots indicate significant single-nucleotide polymorphisms (SNPs) identified through common significant SNPs from three GWAS models, GLM, MLM, and ECMLM. Plots show the GWAS result of the ECMLM model. LD heatmaps were generated for intervals of about 20 Mb including the significantly associated regions. In the lower panel genes present in the ±500 Kb GWAS target regions are shown. (b) GWAS quantile–quantile plot of fruit orientation. The y-axis indicates the observed −Log_10_-transformed *P* values and the x-axis indicates the expected −Log_10_-transformed *P* values. (c) Boxplot analysis of GWAS results from the three highly significant SNPs (Chr 10, Chr 11, Chr 12) for fruit orientation. Each A/B genotype represents the SNPs in three significant associated GWAS regions. Chromosome numbers of each genotype were designated with A/B genotype on the horizontal axis of the boxplot graph. Numbers above the graph indicate the number of accessions for each genotype. For phenotypic analysis (y-axis), a low number represents the erect phenotype; a high number represents the pendent phenotype.

We performed a GWAS for fruit orientation using an association panel of 288 pepper accessions and the high-quality Dempsey v1.0 reference genome of this study. We used PepperSNP16K array SNP data [35] and compared it to the Dempsey v1.0 reference. GWAS identified 28 SNPs associated with the fruit orientation trait, with most significant associations detected on chromosomes 10, 11, and 12 of the newly assembled ‘Dempsey’ genome (Fig. 2a,b; Supplementary Fig. S2, Supplementary Dataset S2, Supplementary Dataset S3). The most significant SNP on chromosome 12 (UC_Pep_Genomic_6969647) explained over 20% of the phenotypic variation (PVE) of the trait, and was located 1.8 kb from DEMF12G21950, encoding a protein with a zinc-binding in the reverse transcriptase (zf-RVT) domain (Pfam domain: PF13966) (Supplementary Dataset S1). A previous GWAS consisting of 196 diverse pepper accessions detected a significant association in this region as well [36], suggesting that DEMF12G21950 is a strong candidate gene for controlling pepper fruit orientation. In addition, we generated a boxplot for visualization of correlation between the genotype and phenotype across the association panel of 288 accessions for the three most significant SNPs from chromosome 10 (UC_Pep_Genomic_5119667), chromosome 11 (UC_Pep_Genomic_7186095), and chromosome 12 (UC_Pep_Genomic_6969647) (Fig. 2c). Most of the erect peppers carried the B haplotype on chromosome 12, while the contrasting pendent phenotype was associated with haplotype B on pepper chromosome 10 or 11. These results suggest that a factor on chromosome 12 is required for erect fruit phenotype, while factors on chromosome 10 and 11 are acting as putative repressors of erect fruit phenotype.

### Genomic information of pan-genome related pepper accessions

To construct a pepper pan-genome, we selected 11 additional *C. annuum* cultivars, ‘CV3’, ‘ThaiHot’, ‘Maor’, ‘I19–702-1’, ‘LaMuyo-01’, ‘PG1’, ‘UCD10X’, ‘Perennial’, ‘CC-090’, ‘CC-260’, and ‘MR’, which represented much of the genetic diversity among the 288 accessions of the *C. annuum* core collection, as reflected by their phylogenetic relationships (Supplementary Fig. S3). Pepper cultivars, ‘CV3’ and ‘ThaiHot’ were sequenced using a linked-read sequencing approach (to ~100× coverage), and ‘Perennial’, ‘CC-090’, ‘MR’, and ‘CC-260’ were sequenced using Nanopore (Oxford, UK) sequencing (~30× coverage with Illumina HiSeq 150 bp reads) (Table 1). We performed reference-guided genome assembly for each pepper accession. The scaffold N50 sizes of 11 pepper genome assemblies ranged from 3.69 to 260.6 Mb (Table 1). The sizes for the final genome assemblies ranged from 3.03 to 3.22 Gb, with an average of 96.3% scaffolds anchored to chromosomes (Table 1). Consistent with this very high percentage of anchored scaffolds, we obtained similar numbers of genes in each pepper genome. The annotation of all pepper genomes identified between 35 018 and 39 938 gene models (Table 1), which was close to the 34 903 genes annotated in the reference genome of the ‘CM334’ pepper (v.1.55) [25]. Moreover, benchmarking universal single-copy ortholog (BUSCO) analysis (Embryophyta odb10) revealed >96% completeness for all pepper genome assemblies reported here (Table 1).

**Table 1:** Statistics of pepper genome assemblies

**Name**	**Contig N50** **(Kb)**	**Scaffold N50 (Mb)**	**Assembly size (Mb)**	**Anchored size (Mb)**	**Repeat contents (%)**	**Gene number**	**Completeness (%, BUSCO)**	**Notes** ^a^
Dempsey	18 322	260.6	3053.5	3044.2	88.06	39 262	97.7	SNU
CV3	5664		3033.1	2913.4	85.72	37 007	98.1	SNU
ThaiHot	8324		3060.2	2900.2	86.64	36 842	97.5	SNU
Maor	78	140.4	3122.0	3069.9	87.64	39 645	98.3	NRGene
I19–702-1	89	229.9	3085.5	3039.1	86.40	39 605	98.4	NRGene
LaMuyo-01	81	132.5	3082.5	3034.5	86.35	39 487	98.2	NRGene
PG1	83 989	174.6	3115.3	3065.5	86.34	39 938	98.3	NRGene
Perennial	97 123	96.9	3038.2	3029.3	88.23	37 876	97.3	SNU
UCD10X	123	3.69	3220.2	2672.1	84.91	35 018	96.4	UC Davis
CC-090	186 906		3064.7	3056.2	88.42	38 361	98.4	SNU
CC-260	135 005		3071.5	3061.4	88.41	38 547	98.3	SNU
MR	106 734		3062.4	3053.7	88.43	38 037	98.1	SNU

a Involved in sequencing and *de novo* assembly.

Repetitive DNA made up >84% of each pepper genome, ranging from 84.9% to 88.4% (Table 1). The most abundant repetitive sequences were retrotransposons (RTs) (an average of 69.6% of 12 pepper genome assemblies), of which RTs with long terminal repeats (LTR RTs) accounted for an average of 66.05% of the pepper genome assemblies (Table S6). The most frequent RT belonged to the *Gypsy* family, covering about 56.0% to 57.1% of the pepper genome and representing 60.2% of all repeat elements. The second most abundant RTs were *Copia* elements, making up 6.1–6.2% of the pepper genomes and 6.2% of all repeat elements (Table S6). Long interspersed nuclear elements (LINEs), non-LTR RTs, and short interspersed nuclear elements (SINEs) represented another 3.6–3.65% of the pepper genomes (Table S6). DNA transposons, also called class II elements (DNA transposons and *Helitrons*), accounted for 8.7–9.2% of the genome (Table S6).

### Gene family analysis of 12 pepper genomes

**Figure 3:**
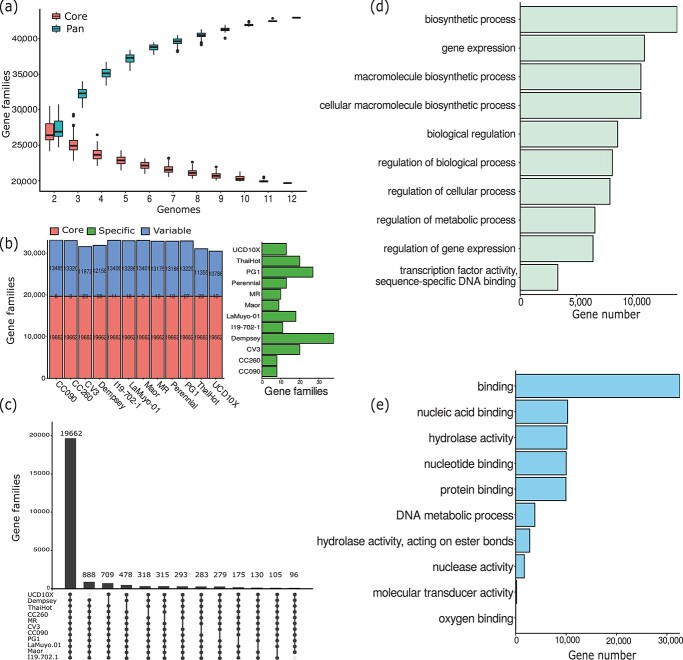
Pan-genome analysis of 12 *C. annuum* accessions. (a) Number of average core and pan-gene sets identified through ortholog analysis of pan-genomes. Pink, core gene sets; cyan, pan-gene sets. The incorporation of additional genomes, following the inclusion of 11 pepper genomes, leads to a minor increase in pan-genome size. (b) Number of core, non-core, and specific genes across multiple accessions. Enlarged graph of the specific gene families is displayed in right panel. (c) Number of core gene orthologs (present in at least 11 genomes) and their distributions across accessions. Black dots at the bottom of the graph indicate presence in the respective genomes. (d) Gene Ontology (GO) term enrichment analysis results for the top 10 ranked GO terms in the core gene set. (e) GO term enrichment analysis results for the top 10 ranked GO terms in the non-core gene set.

A search for gene families classified all genes annotated across the 12 pepper genomes into 42 972 gene families (Fig. 3a). The total number of gene families increased with each addition of a genome, but the number of core- and pan-gene families were nearly flattened when 10 or more genomes were added, reflecting the good representation of the pan-gene landscape offered by the 12 selected pepper accessions. Based on the genome size of the Dempsey v1.0 assembly presented here (3.05 Gb), cumulative lengths of 79.39–149.70 Mb additional genomic sequences (including PAVs and CNVs) were found in the other pepper genome sequences. Of the 42 972 gene families, we classified 19 662 (276 220 genes) as core gene families (present in 12 genomes) (Fig. 3b and c). Another 23 115 gene families (178 616 genes) were non-core (present in 2–11 accessions) (Fig. 3b), which represented about 39.19% of all genes in the pan-genome (Fig. 3b). The percentage of specific genes ranged from 0.05 to 0.68% in the different genomes.

Gene Ontology (GO) term enrichment analysis on core and non-core genes indicated that core genes were most often enriched for GO terms related to biosynthetic process, gene expressions, biosynthetic process, biological regulation, while non-core genes appeared to be enriched for nucleotide/protein binding, hydrolase activity, DNA metabolic process, and nuclease activity (Fig. 3d,e).

### Genomic SVs between pan-genome pepper lines

**Figure 4:**
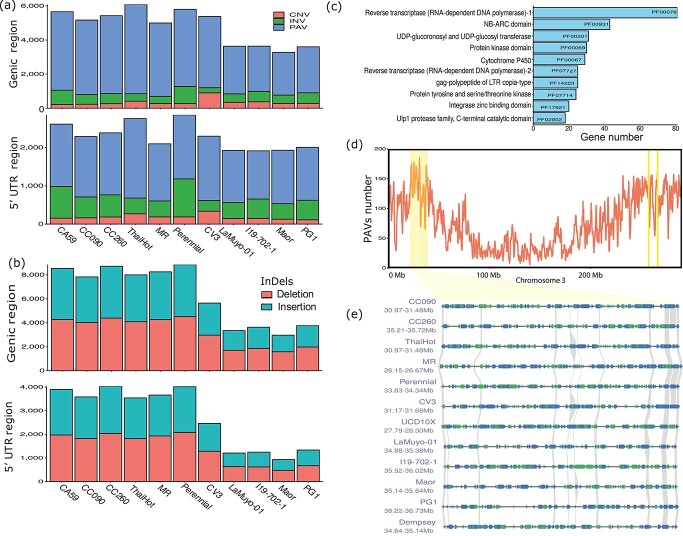
Summary of structural variants (SVs) across the *C. annuum* pan-genome. (a) Summary of presence–absence variants (PAVs), copy-number variants (CNVs), and inversions (INVs) in genic and 5′ regions (within 1 kb upstream of the start codon) of the 12 pepper accessions. (b) Summary of small insertion/deletions (InDels) in genic and 5′ UTR regions (within 1 kb upstream of the start codon) of 12 pepper accessions. (c) Top 10 ranked Pfam domains from PAV hotspot regions (1 Mb window size). (d) Representative PAV hotspot regions for pepper chromosome 3 (The longest spanned fragments in hotspot region). The red line indicates the number of PAVs in 1 Mb sliding windows. Yellow shading indicates PAV hotspot regions in chromosome 3. (e) Microsynteny analysis of the PAV hotspot region on chromosome 3. Gray lines indicate the homology between the 12 pepper genomes. Genes on the positive strand are shown in blue; genes on the negative strand are shown in green.

To identify SVs, we aligned the assembled genome sequences of the 12 pepper accessions including recently published high quality pepper genome, ‘CA59’ [37], to the pivot genome, Dempsey v1.0. The alignments showed good co-linearity between pan-genome individuals and the ‘Dempsey’ reference genome (Supplementary Fig. S4). We determined that an average of 94.9% (93.09–96.36%) of the ‘Dempsey’ genome exhibits a syntenic relationship with each of the other 11 pepper genomes (Table S7). Despite the high percentage of co-linearity, about 150 Mb of genomic sequence on average did not agree between Dempsey v1.0 and the other pepper genomes. We identified 16 032 inversions (INVs) ranging from 1000 bp to 3.75 Mb and distributed across all 12 chromosomes. Together, these INVs harbored approximately 6527 genes in ‘Dempsey’, of which a large fraction (2732 genes) were annotated with functions related to protein binding (GO:0005515) (Supplementary Dataset S4). We also obtained 12 322 CNVs and 222 159 PAVs from the alignment of the 11 pepper genomes. For each pan-genome member compared to ‘Dempsey’, we identified 300–6381 INVs, 1898–5238 PAVs, and 219–906 CNVs that overlap with protein-coding genes (Fig. 4a). In 5′ untranslated regions (UTRs), we detected 277–6202 INVs, 637–2077 PAVs, and 111–357 CNVs (Fig. 3b; Table S8). The UCD10X genome assembly stood out with many more INVs relative to ‘Dempsey’ than the other 11 pepper genomes, which might reflect the quality of the UCD10X assembly and the unknown orientation of its genome contigs. In addition to INVs, we detected an average of 131 972 InDels (with sizes no more than 50 bp) (Fig. 4b) and an average of 7 279 285 SNPs across the pan-genome. PAVs were usually enriched in distal regions (Fig. 1c; Supplementary Fig. S5); we also identified genomic regions as potential hotspots for PAVs (Supplementary Fig. S5; Supplementary Dataset S5). The hotspot region on chromosome 3 harbored many genes involved in stress responses (NB-ARC, protein kinase, and cytochrome P450) and was characterized by a complex genetic architecture (Fig. 4c–e).

We observed similar distribution patterns for PAVs within hot and sweet peppers genomes, however sweet peppers exhibited lower number of PAVs in 5′ UTRs than hot peppers, which had more InDels than sweet peppers (Fig. 4a,b), indicating the complex and distinct genomic architecture of hot and sweet peppers. Combining all PAVs from the 12 pepper genomes, we constructed a pan-genome by following a graph-based strategy [38, 39].

### Validation of known SVs in constituent accessions of the pan-genome

To test the power of a graph-based pepper pan-genome for the detection of genetic variants associated with agricultural traits, we first focused on the genomic regions harboring the genes *Pun1*, *Capsanthin/capsorubin synthase* (*CCS*), *potyvirus resistance6* (*pvr6*), and *FASCICULATE* (*FA*) to search for variation that might correlate with the known phenotypes of the pan-genome accessions [2, 40–42] (Table S9 and S10). Non-pungent peppers carry a deletion in the 5′ region of *Pun1*; we identified a deletion within the coding region of *Pun1* in the genomes of all sweet peppers from this study (‘Dempsey’, ‘LaMuyo-01’, ‘I19–702-1’, ‘PG1’, and ‘Maor’) (Supplementary Fig. S6; Table S10). All sweet pepper genomes had a 2.57-kb deletion spanning the promoter, 5′ UTR, and first exon of *Pun1*, whereas we detected no such deletion in the hot pepper genomes (‘Perennial’, ‘ThaiHot’, ‘MR’, ‘CC260’, ‘CC090’, and ‘CV3’). For the UCD10X genome, an F_1_ hybrid between pungent and non-pungent pepper accessions, the non-pungent allele types were detected in the chromosome 2 while the other allele was detected on the other haplotig [28]. *CCS* is an important gene associated with variation for fruit color in pepper. The genome of accession ‘I19–702-1’, characterized by yellow fruits, showed a large deletion of 4430 bp in the *CCS* gene resulting in a non-functional *CCS* allele. The *pvr6* mutation is a recessive allele that confers resistance to potyvirus and was originally identified in the cultivar ‘Perennial’. The causal mutation was mapped to a 83-bp deletion in the first exon of *Eukaryotic initiation factor 4E isoform* (*eIF(iso)4E*) [41]. The wild-type *Pvr6* allele lacking this 83-bp deletion is present in the genomes of potyvirus-susceptible pepper accessions. We detected the truncated nonfunctional *pvr6* allele in the ‘CV3’ pepper genome based on our pan-genome study (Supplementary Fig. S6). Nonfunctional alleles of *FA* are responsible for the *fasciculate* fruit clusters mutation [42]. We discovered a 428-bp deletion spanning the second exon of *FA* in the genome of the accession ‘ThaiHot’ (Supplementary Fig. S6), which bears fasciculate fruit clusters. Together, these results highlight the utility of a pan-genome for the identification of PAVs in pepper genomes, which will constitute a useful resource for pepper molecular breeding research.

### SVs analysis of hot and blocky sweet peppers using the pan-genome PAVs

**Figure 5:**
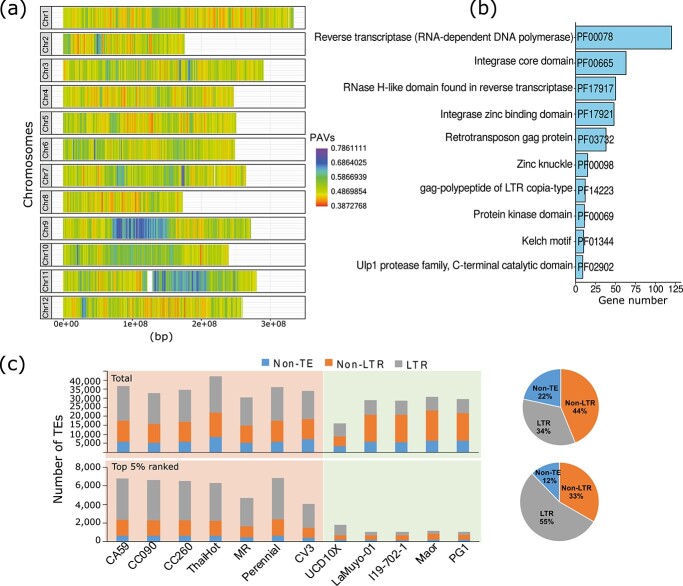
In-depth genomic analysis of structural variation between hot and sweet peppers. (a) Frequency of the presence–absence variants (PAVs) along the 12 pepper chromosomes represented as a heatmap according to the color scale shown to the right (lowest values in red, highest values in purple). Frequency was calculated in sliding windows (1-Mb window, 200-kb increment). (b) Top 10 ranked Pfam domains in the top 5% of PAV frequency regions. (c) Comparative analysis of PAVs associated with transposable elements (TEs). “Total” represents whole genome PAVs/TEs generated from the pan-genome analysis. “Top 5% ranked” represents top 5% ranked PAV frequency regions between hot and sweet peppers.

Using the pepper pan-genome, we calculated the frequency distribution of PAVs along the chromosomes of hot and sweet pepper accessions using a sliding window approach. Specific chromosomal regions exhibited higher frequencies of PAVs than other regions (Fig. 5a). We selected the top 5% genomic regions with high PAV frequency (covering 230.6 Mb size) that differentiate hot and sweet peppers (Fig. 5a; Supplementary Table S11). These regions contain many genes of repeat domains (PF00078, PF00665, PF17917, and PF17921) (Fig. 5b). In addition, we compared the whole genome distribution of transposable elements (TEs) with respect to the PAVs distribution (Fig. 5c). Both whole genome and top 5% ranked region comparisons revealed proportionally higher number of PAVs in genomic areas with high TEs (Figure 5c). However, in case of top 5% enriched PAVs genomic regions, higher number of PAVs were largely correlated with relatively higher number of LTRs presence, suggesting a key role for LTRs in larger genomic variations, such as PAVs.

**Figure 6:**
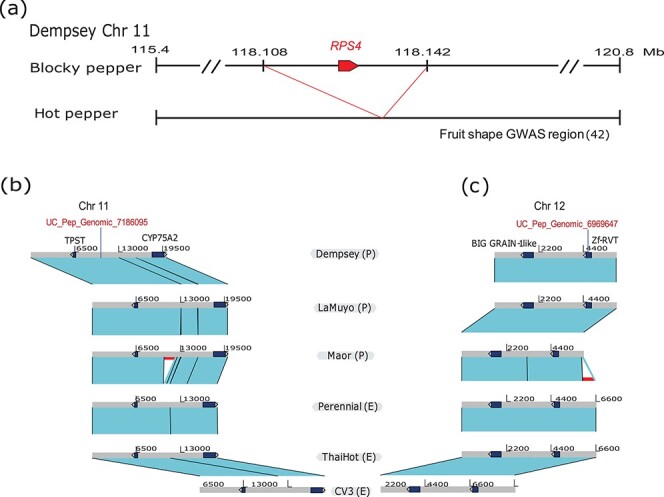
Identification of PAVs in candidate gene regions. (a) Schematic diagram of putative fruit shape-associated genes and associated PAVs on chromosome 11. Previously reported regions associated with fruit shape variation were projected onto the ‘Dempsey’ genome by BLAST. An ~34-kb deletion differentiating sweet and hot peppers harbored one candidate gene, *RPS4* (*Ribosomal protein S4*). (b) and (c) Comparison of significant presence–absence variants (PAVs) in previously detected associated GWAS regions on Chr 11 and Chr 12. Cyan boxes indicate the syntenic regions between two genomes. Solid dark blue boxes with arrowheads represent protein-coding genes within the GWAS regions. Red thick lines indicate the PAVs in GWAS regions. Genotyping-by-sequencing GWAS SNP information is shown above the gene description in red text. TPST: Tyrosylprotein sulfotransferase. CYP75A2: Cytochrome P 750A2. zf-RVT: Zinc finger domain containing reverse transcriptase.

Furthermore, to explore genomic clues for fruit-related traits in pepper, we investigated the regions with high PAV frequency within the GWAS regions for fruit shape [1, 43]. All previously detected GWAS regions on chromosome 11 associated with fruit shape traits were included in ±1 Mb region of the top 5% of regions with high PAV frequency, as expected (Supplementary Table S12). We also detected a single PAV located in the GWAS region on chromosome 11 associated with fruit shape. This region harbored a gene encoding Ribosomal protein S4 (RPS4) (DEM11G09440), which was absent in hot pepper accessions (Fig. 6a), suggesting a possible role for *RPS4* in pepper domestication. Additionally, we detected PAVs in fruit shape related candidate genes in 5′, coding, and intergenic regions from the high PAV regions (top 5%) on chromosome 2, 6, 7, 10, 11, and 12 (Supplementary Table S13).

Using PAV information from the graph-based pan-genome, we identified PAVs co-segregating with fruit orientation in the 5′ UTR of pepper genes encoding tyrosylprotein sulfotransferase (TPST) (400 bp upstream) on chromosome 11 and a putative zinc-finger including ribonuclease H protein (zf-RVT) (1.1 kb upstream) on chromosome 12, both located within the GWAS significant regions (Fig. 6b). Notably, the genomes of the accessions with erect fruits (‘Perennial’, ‘ThaiHot’, ‘CV3’, and ‘MR’) showed a 572-bp deletion in the promoter region of the *zf-RVT-like* gene. These results demonstrate the broad utility of the newly assembled ‘Dempsey’ reference for comparative genomics and genetic dissection of agriculturally important traits.

## Discussion

Genome SVs represent an important component of genetic diversity in plants that have gained substantial interest due to their influence on phenotypic variation. In the early days of plant genomic analyses, technological limitations and reference genomes and assemblies of lower quality precluded a comprehensive exploration of SVs, especially in plants with large and complex genomes [44]. With recent improvements in DNA sequencing technologies, as well as with the use of advanced bioinformatics tools and pipelines, the identification and analysis of SVs is becoming increasingly more common and within reach, including for plant genomes [45, 46]. Nevertheless, it is difficult to accurately detect large SVs and their intricate structures based on short-read sequences, simply because the read length is very often shorter than the SV size. Furthermore, short reads pose challenges for the detection and characterization of highly repetitive regions in particular in highly complex genomes such as pepper [47]. Despite numerous improvements in sequencing, whole-genome mapping studies, algorithms, and tools, a high-quality and accurately assembled *Capsicum* genome is lacking, limiting comparative genome-wide SV studies across *Capsicum* species. To fill this gap, we constructed a high-quality genome assembly of the ‘Dempsey’ using a combination of PacBio long-reads, Hi-C, short reads, optical mapping, and classical genetic maps. The final Dempsey v1.0 genome assembly had a contig N50 value of 18.3 Mb.

Compared to existing pepper genome assemblies, the contig N50 value of the Dempsey v1.0 genome assembly is six times greater, and the scaffold N50 value is at least 80 times higher than earlier pepper genomes [25, 28]. In addition, only a small fraction (0.4% or 9.33 Mb) of the Dempsey v1.0 genome remains unanchored, which marks a considerable improvement over previous pepper reference genomes, with unanchored sequences accounting for ~17–20% (or 500–800 Mb) of the entire genome [25, 28]. The final ‘Dempsey’ genome assembly (v1.0) comprises 121 scaffolds, and the 12 pseudochromosomes, which represented 99.6% of the entire assembly, are congruent with multiple pepper genetic maps [30, 34, 35]. To date, pepper genomic studies have had to rely on the reference genomes of hot or F_1_ hybrid (hot × sweet) peppers. The new high-quality ‘Dempsey’ reference genome is the first pepper genome based on a bell-type pepper, which is the main non-pungent pepper type grown worldwide.

In this study, we applied a novel graph-based approach to produce a pepper pan-genome assembly using 12 long- and linked-read-sequenced accessions. Graph-based pan-genomes allow nearly all structural variants to be represented more efficiently, represent a more sustainable approach than linear references, and are useful to find agriculturally important variants at the population level [39, 48]. The pepper pan-genome constructed here based on 12 accessions revealed that 48.67% of pan-genes are core genes, while another 31.47% of genes are non-core. Previous studies suggest that the number of genomes included in the pan-genome construction can greatly influence the pan-genome metrics and analyses [9, 13, 49]. Based on a comparative *de novo* assembly of three divergent rice species, Schatz *et al.* [50] defined 92% of all genes as core genes and only ∼8% as variable genes. Similarly, a pan-genome for cabbage (*Brassica oleracea*) constructed based on the genomes of 8 cultivars determined that ∼56% are core gene clusters, while ∼42% are non-core gene clusters.

Pan-genomes aid in the discovery of genes missing from the original reference genomes, highlighting the significance of sequencing more than one genome to capture the broad genetic diversity of a given species [15, 17]. To-date several reports in pepper have been published using resequencing with short reads and mapping to reference genome, including reports of Kim *et al*. [27], Ou *et al*. [33] and Acquadro *et al*. [29]. In contrast, we present a comparison of structural variants of 11 de novo-assembled genomes using a comprehensive long-read assembly of the cultivar ‘Dempsey’ as the pivot genome. By doing so we describe not only SNP and indel, but also large SVs including PAVs, CNVs, and INVs not captured in resequencing experiments (Supplementary Table S8). In addition, we identified SVs in the pepper pan-genome in the important genes *FA*, *pvr6*, *CCS*, and *Pun1* in agreement with the corresponding phenotypes of the individual accessions. This indicates that PAVs identified in the present study may be a powerful new tool to identify their largely unknown phenotypic manifestations through reverse genetics.

Furthermore, using the graph-based pan-genome defined here, we mapped a PAV within the GWAS region associated with pepper fruit shape [1, 43] and fruit orientation. The GWAS region for fruit shape harbors a gene coding for Ribosomal protein S4 (DEM11G09440), which is exclusively present in blocky peppers, implicating this protein in an unknown mechanism yielding bell- or blocky-type fruits. GWAS identified 28 SNPs associated with fruit orientation on chromosomes 7, 8, 10, 11 and 12 of the Dempsey v1.0 genome, accounting for 7–20% PVE. We noticed that the SNPs highlighted by GWAS map close to *TPST* (and the nearby *Cytochrome P750A2* gene [*CYP75A2*]) on chromosome 11 and to a gene encoding a zf-RVT domain-containing protein and *BIG GRAIN-like* on chromosome 12, associated with fruit orientation (Fig. 6b). However, the physiological roles of these genes are not well established. Homologs to *TPST* are involved in fatty acid metabolism in maize and are thought to play a key role in plant growth and development [51]. The zf-RVT domain is normally found in reverse transcriptases [52]. *BIG GRAIN-like* was previously reported to be involved in auxin transport and to regulate plant growth, development, and grain size in rice [53], making its pepper homolog a likely candidate gene for controlling variation in fruit orientation.

In summary, we developed a high-quality genome assembly for the pepper ‘Dempsey’ by combining long-read and short-read sequencing technologies. Using the new ‘Dempsey’ genome as a pivot reference, we constructed a graph-based pan-genome, detected large structural variants across the genome in *C. annuum* accessions, and identified important genetic variants for the determination of hot or sweet pepper fruits. Overall, the high-quality ‘Dempsey’ reference genome and *C. annuum* pan-genome provide an important resource for facilitating fundamental research and genetic improvement of the pepper crop.

## Materials and methods

### Plant materials and DNA extraction

*C. annuum* cultivars used for genome analysis were ‘Dempsey’, ‘CV3’, ‘ThaiHot’, ‘Maor’, ‘I19–702-1’, ‘LaMuyo-01’, ‘PG1’, ‘UCD10X’, ‘Perennial’, ‘CC-090’, ‘CC-260’, and ‘MR’. Four pepper genome sequences were provided by collaborators from the NRGene Consortium: ‘Maor’, The Volcani Center, Israel; ‘I19–702-1’, Pilpel Seeds, Israel; and ‘LaMuyo-01’, BASF-Nunhems, USA; ‘PG1’, Top Seeds, Israel. The genome sequence of ‘UCD10X’ was provided by the University of California, Davis, USA. High-molecular-weight genomic DNA was obtained from the cultivars ‘Dempsey’, ‘CV3’, ‘ThaiHot’, ‘Perennial’, ‘CC-090’, ‘CC-260’, and ‘MR’ by isolating nuclei from young leaves as described by Zhang *et al.* [54]. Genomic DNA was then extracted from these nuclei using the Qiagen MagAttract HMW DNA Kit (Cat. no. 67563).

### Construction of the pivot reference genome for the ‘Dempsey’ cultivar

High-quality genomic DNA for the ‘Dempsey’ cultivar was used for sequencing on the PacBio Sequel platform using CLR mode sequencing method. Sequencing was performed at DNAlink company (Seoul, Korea). *De novo* assembly was conducted using FALCON-Unzip assembler using default options with the exception of a length cut-off of 20.3 kb [55]. The FALCON-Unzip assembler and Arrow consensus algorithm were used to polish and generate primary PacBio *de novo*–phased diploid contigs. For error correction, primary PacBio contigs consisting of haplotigs were used as a reference to align Illumina NovaSeq data (80× coverage) using BWA version 0.7.10 and GATK version 3.5. For scaffolding the PacBio contigs using Hi-C technology, a Dovetail Hi-C library was prepared according to the manufacturer’s instructions using a Dovetail™ Hi-C Kit (Dovetail Genomics, Chicago, IL, USA). The final libraries were sequenced on an Illumina NovaSeq 6000 system as 150-bp paired-end reads. Hybrid scaffolding was performed using HiRise software with Hi-C data. Optical mapping was performed using Irys optical mapping technology (BioNano Genomics, San Diego, CA, USA). DNA was labeled at DLE-1 (CTTAAG) sites using the IrysPrep kit. Labeled DNA samples were loaded onto IrysChips and run on the Irys imaging instrument Saphyr (BioNano Genomics). The IrisView software package was used to produce BioNano genome maps. BioNano genome maps were validated with two independently generated genome maps, BioNano + PacBio and BioNano + PacBio + Hi-C maps; any inconsistency between maps was corrected using genetic maps. To construct pseudomolecules, ALLMAPS software [56] was used to combine the four previously reported pepper genetic maps: a ‘Perennial’ × ‘Dempsey’ intraspecific *C. annuum* bin map [30], a ‘NuMex RNaky’ × ‘BG2814–6’ interspecific map (*C. annuum* × *C. frutescens*, FA) [34], an ‘Early Jalapeno’ × ‘CM334’ intraspecific *C. annuum* bin map (NM) [34], and a ‘Tabasco’ × ‘P4’ interspecific Illumina array map [35]. For the two interspecific maps, the known translocations between chromosomes 1 and 8 were curated manually [28]. Information from these combined genetic maps was used to build and anchor the pseudomolecules of the ‘Dempsey’ genome assembly. Scaffolds spanning two linkage groups were manually corrected according to the ‘Perennial’ × ‘Dempsey’ genetic map. To manually fill gaps in the ‘Dempsey’ genome, PCR primers were designed to anneal 500 bp upstream and downstream of a given gap sequence. Amplified single-copy PCR fragments were sequenced by Sanger dideoxy sequencing (Macrogen, Seoul, Korea).

### Reference-guided chromosome construction

Chromosome sequences of *C. annuum* chromosome were obtained through DeNovo PanMagic analysis in NRGene [57]. Additionally, RagTag v2.0.1 was used for merging the Nanopore contig sequences of ‘Perennial’, ‘CC-090’, ‘CC-260’, and ‘MR’ in the order of the pivot genome [58].

### Gene prediction and functional annotation

First, to increase the accuracy of pepper genome annotation, pepper transcript dataset from NRGene was aligned to each pepper genome and used this alignment file for a hintsfile in Augustus annotation. Augustus 3.2.3 were used for gene prediction in 12 pepper genomes with the species option as tomato, which was closest Solanaceae crops with *Capsicum* species. Raw gene annotation results were filtered out by aligning predicted genes against previous published pepper peptide sequence datasets (identity >70%; coverage >70%).

### SV variant analysis

MUMMER v4.0.0 was used for genome-to-genome alignment (nucmer –g 1000 –c 90 –l 64), and PAVs were identified using Assemblytics with default options [62, 63] using ‘Dempsey’ as a reference genome. PAVs were also identified using another pipeline, minimap2 (−ax asm5 —eqx) and SyRI v1.5 (−k –F -S) [64, 65]. Because of the higher sensitivity of deletions than insertions in genome alignment step7, reciprocal genome alignment was also performed. Subsequently, PAVs were extracted from the Insertion/Deletion calls from Assemblytics and Insertion/Deletion/HDR (Highly divergent region) calls from the SyRI results. Common calls between Assemblytics and SyRI were considered for PAVs. For CNV identification, Assemblytics calls annotated as “Deletion” or “Tandem_contraction” were identified in pairwise and reciprocal genome alignments. SyRI calls annotated as “DUPAL”, “HDR”, or “CPL” were identified in both pairwise and reciprocal alignment. Then, common variants of Assemblytics and SyRI analyses were considered as CNVs. For small indel extraction, similar approach to the PAV extraction was performed with delmit option set to 5–49 bp. For INVs and SNPs, SyRI calls (INVAL and SNP) were used for downstream analysis.

We used ‘Dempsey’ as the reference genome and nonredundant PAV variant calling format (VCF) files to construct the pan-genome graph using construct module as implemented in the vg toolkit program [38].

### Construction of the phylogenetic tree of the *C. annuum* clade

To infer the phylogenetic relationship of 12 accessions in the pepper pan-genome, a phylogenetic tree was constructed using GBS SNP data from a pepper core collection [23]. First, *C. annuum* biallelic GBS SNPs were filtered using vcftools (—remove-indels —maf 0.005 —minDP 3 —minGQ 20 —min-alleles 2 —max-alleles 2) and merged with the pan-genome SNP dataset. Then, the resulting merged SNP data were filtered using vcftools (—max-missing 0.5) and used to construct a phylogenetic tree. Iqtree v1.6.2 (−bb 1000 –m GTR + ASC) was used to generate a phylogenetic tree using a phylip-formatted SNP dataset [59]. An admixture plot was generated using ADMIXTURE with the k = 3 option [60].

### Pan-genome analysis

To generate core and variable gene lists for the *C. annuum* pan-genome, predicted genes were clustered using OrthoFinder v2.27 (−M msa) [61]. According to previous pan-genome analysis in rapeseed (*Brassica napus*) [13], gene clusters were classified as core (genes present in 12 genomes), non-core (genes present in two to 11 genomes), and specific (genes present in only one genome). Core and variable gene clusters were visualized using custom R scripts and TBTools [62]. Enrichment for GO terms among core and non-core genes was analyzed using AgriGO version 2^62^ and visualized using custom R scripts.

### Repeat annotation

To annotate repeats in the ‘Dempsey’ genome, a combination of *de novo* and homology-based searches were performed using RepeatMasker and RepeatModeler (http://www.repeatmasker.org/). First, custom repeat libraries were generated using RepeatModeler. Then, RepeatMasker was employed to identify repeat sequences in the pepper genome against all repeat libraries from *C. annuum* custom repeat libraries.

### Identification of PAV hotspot regions

Total PAVs generated from pan-genome analyses were used for downstream analysis. PAV density was calculated by a sliding window approach (1-Mb window with 200-kb increments) using custom Python scripts; the top 1% were considered as PAV hotspot regions, with overlapping regions merged manually. These regions were compared and visualized using the MCscanX tool [64] and Artemis Comparison Tool (ACT) [65].

To calculate distinctive regions between hot and sweet peppers, the relative PAV frequency was manually calculated by the gap between the average PAV number in hot peppers (‘CC-090’, ‘CC-260’, ‘Perennial’, ‘ThaiHot’, ‘MR’, and ‘CV3’) and that of sweet peppers (‘LaMuyo-01’, ‘I19–702-1’, ‘PG1’, and ‘Maor’). The frequency at each PAV position was visualized using a sliding window approach generated from custom Python scripts (1-Mb window with 200-kb increments). The top 5% ranked PAV frequency regions were considered high PAV frequency regions between hot and sweet peppers. Then, a previously reported GWAS region associated with fruit shape [1, 43] was compared to the high PAV regions above, and overlapping regions were used to find significantly different regions between hot and sweet peppers.

### Chip genotyping

The PepperSNP16K array [35] was used to genotype 288 *C. annuum* complex accessions from the pepper core collection (Illumina, Inc., San Diego, California, USA). To locate chip marker positions in the ‘Dempsey’ genome, ~100 bp of sequence flanking either side of each chip marker (SNP) was searched in the ‘Dempsey’ genome by BLAST. Markers that aligned at four or more sites, as well as markers that were not aligned to ‘Dempsey’, were eliminated. By filtering markers with minor allele frequencies below 0.05 and call rates below 90%, we obtained 13 146 markers.

### GWAS

GWAS was conducted using the genotypic data obtained by array and BLINK models, which were provided by the R package “GAPIT3” [66]. The following approach was used to designate the phenotype for fruit position: 1 for erect position, 3 for intermediate phenotype between erect and pendent position, and 6 for pendent position. The average measurements over a 3-year period from 2015 to 2018 were utilized as phenotypic data. The Bonferroni correction method was used to compute the adjusted *P* value by dividing statistical significance by the number of markers to avoid error accumulation. Markers below the Bonferroni-adjusted *P* value (<0.05) were considered significant.

## Supplementary Material

Web_Material_uhac210

## Data Availability

The data that support the findings of this study are available in the supplementary material of this article. The *C. annuum* whole-genome sequence data have been deposited at NCBI under the accession number PRJNA706772 (BioSample IDs: SAMN31130554-SAMN31130564). The PAV datasets and codes were deposited in GitHub (https://github.com/JONGHOLEE-myrrh/Pangenome).
