# Supplementary material for: High-quality chromosome-scale genomes facilitate effective identification of large structural variations in hot and sweet peppers
Source: Hortic Res. 2022 Sep 19;9:uhac210. doi: 10.1093/hr/uhac210 (PMC9715575; doi:10.1093/hr/uhac210)
Supplement: Web_Material_uhac210 [file web_material_uhac210.zip › Supplemental Information HR_R1.docx]

**
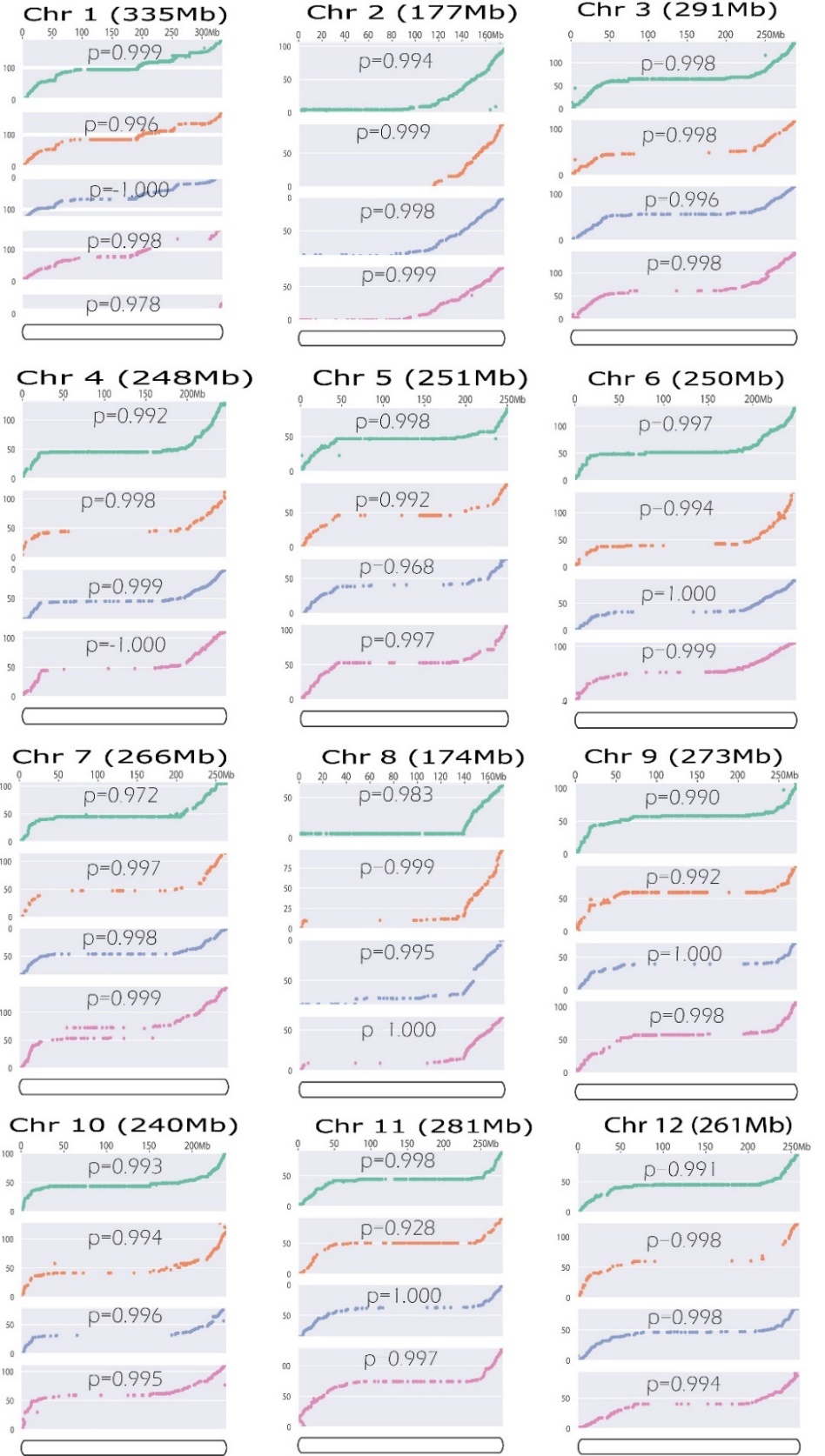
**

**Fig. S1.** Scatterplot representation of the physical position (x-axis, Mb) and the genetic distance (y-axis, cM) of markers along the 12 pepper chromosomes. The *P* value on each scatterplot represents the Pearson’s correlation coefficient. We used four pepper genetic maps, FA3200929 (green), NM.200929 (orange), PD.1st.200929 (blue), and array.200929 (pink), with different weights to reconstruct all ‘Dempsey’ chromosomes.


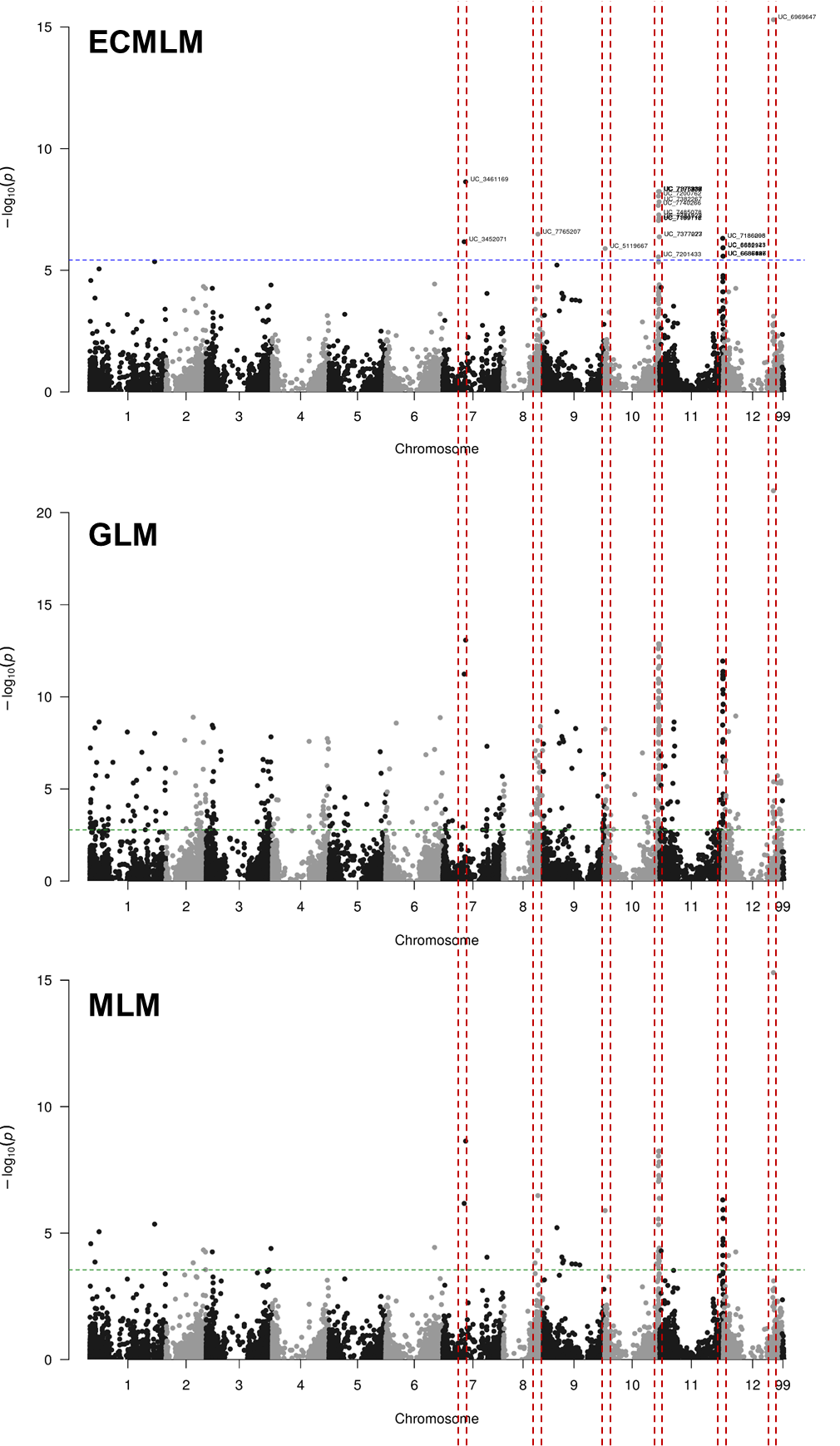


**Fig. S2.** GWAS analysis of fruit orientation using three models, ECMLM, GLM, MLM. Green horizontal lines represent the threshold calculated by FDR rate. Common regions were designated by red vertical lines. Chromosome 99 represents unanchored scaffolds.

**
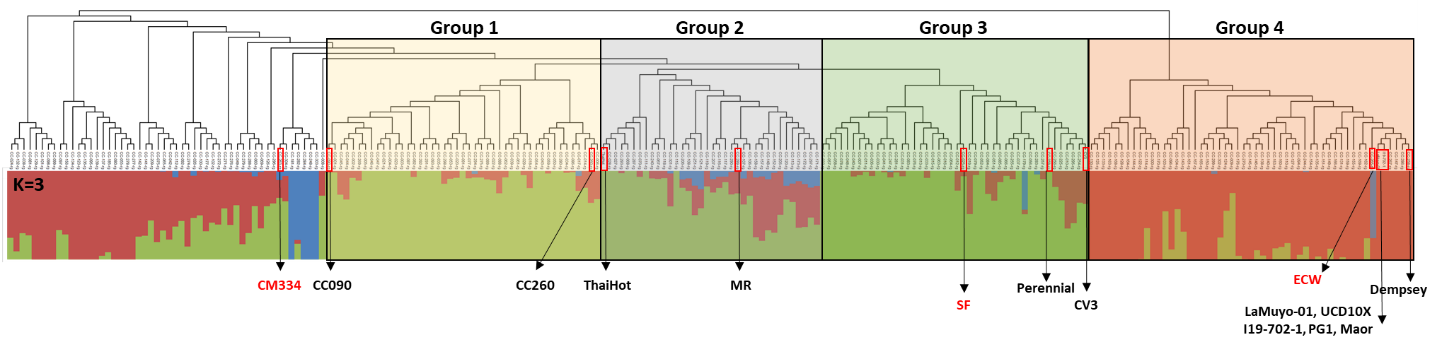
Fig. S3.** Phylogenetic analysis of *Capsicum annuum* accessions from the pepper core collection. Subgroups were divided by clustered phylogenetic nodes. Admixture analysis was performed with three subpopulations (K=3). Previously published pepper genomes (CM334, SF, and ECW) are indicated in red font.


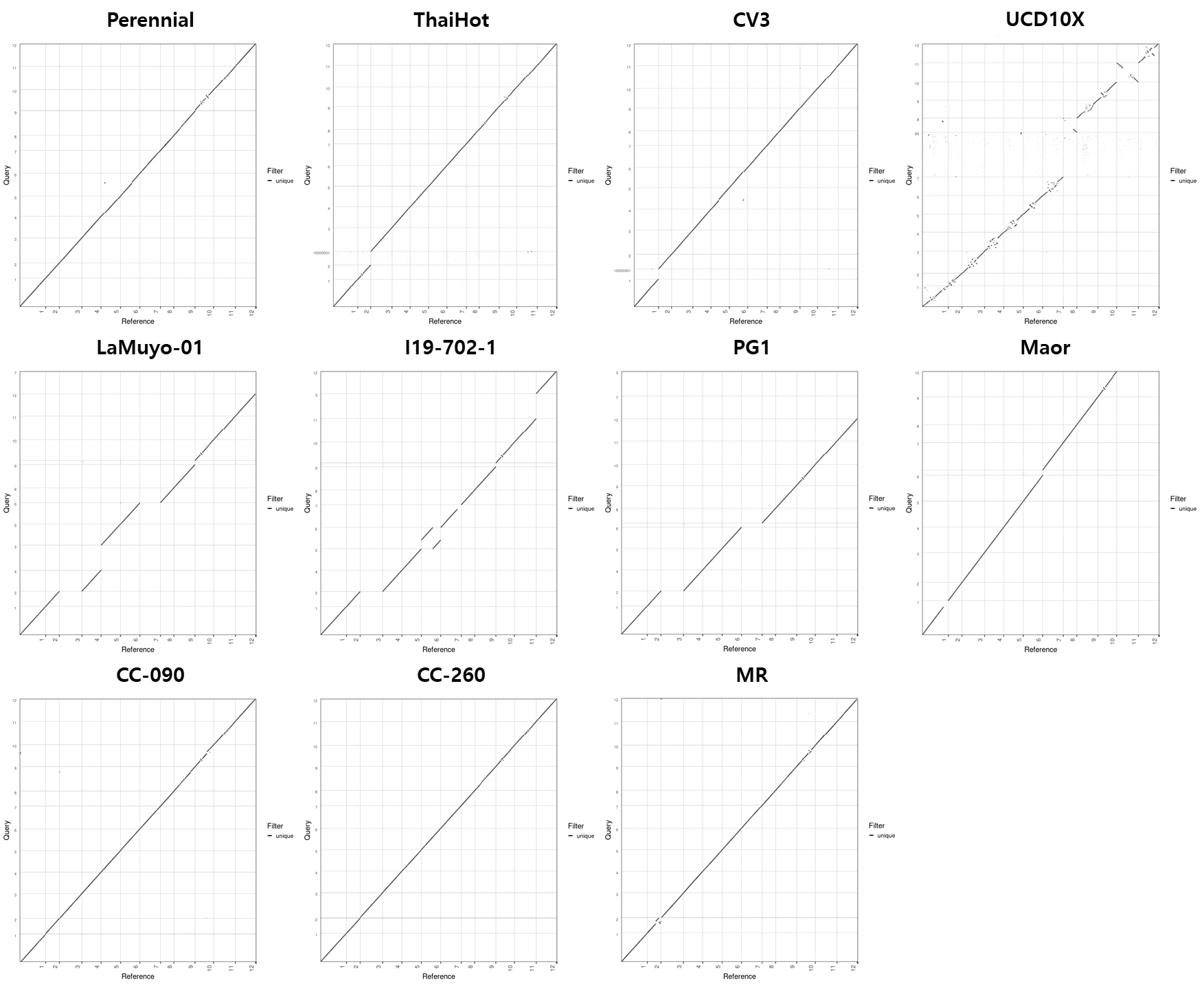


**Fig. S4.** Dot plot analysis of 11 pepper genome sequences (CV3, I19-702-1, LaMuyo-01, Maor, Perennial, PG1, ThaiHot, UCD10X, CC-090, CC-260, and MR) using the Dempsey genome as a pivot reference. Dot plot analysis was carried out for the 11 pepper genome sequences (x- axis) and Dempsey (y-axis) with the DOTTER program.


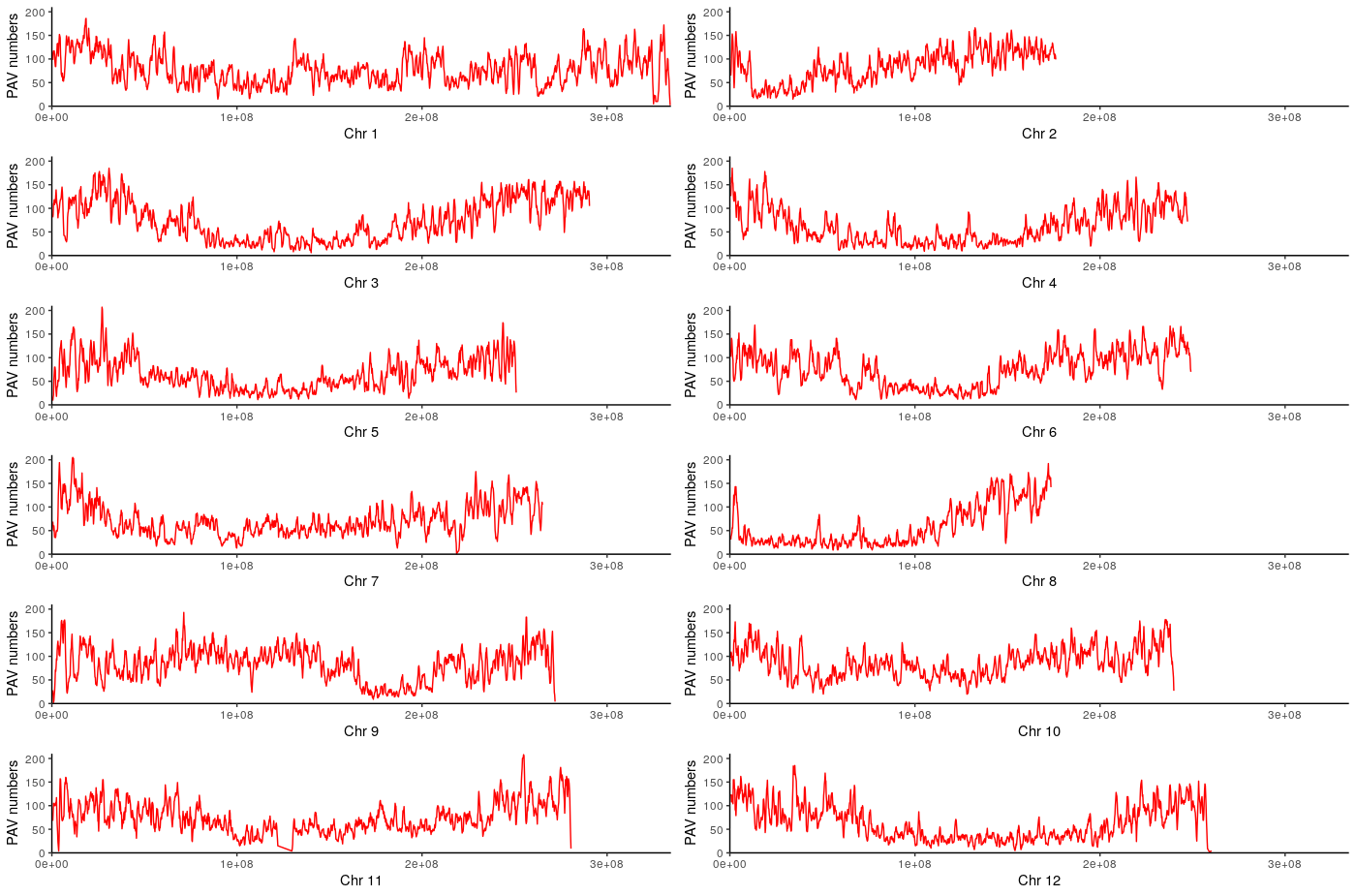


**Fig. S5.** PAV density plots along the twelve pepper chromosomes. The y-axis represents the number of PAVs in 1-MB sliding windows with 200-kb steps. The x-axis represents the physical position of the center of the sliding windows on each chromosome.


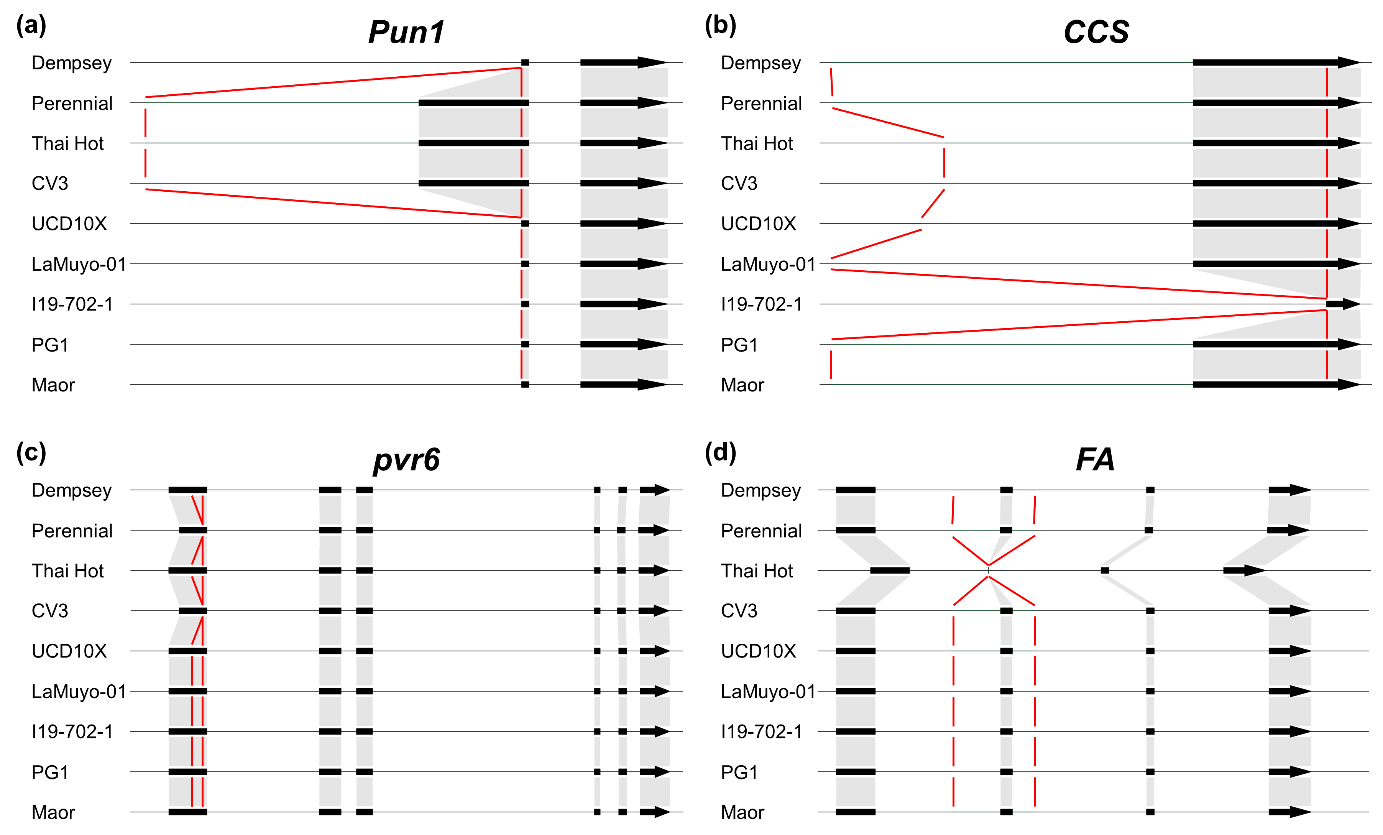


**Fig. S6.** Schematic representation of structural variations detected in agronomically important genes in pepper lines. **(a)** *Pun1*. **(b)** *Capsanthin-capsorubin synthase* (*CCS*). **(c)** *pvr6*. **(d)** *FA*. Lines represent introns, black rectangles represent exons, and arrows indicate direction of transcription. Genes displaying presence–absence variation are shown as red lines. Gray boxes indicate conserved exons among pepper genomes.

**Table S1.** Summary statistics of the Dempsey genome assembly.

|  | Number of contigs (scaffolds) | Mean length (Mb) | N50 size (Mb) | Maximum length (Mb) | Total assembly length (bp) | No. of genes annotated | No. of gaps |
| --- | --- | --- | --- | --- | --- | --- | --- |
| Contigs from PacBio assembly | 500 | 6.07 | 18.319 | 85.419 | 3,035,058,527 | - | - |
| Contigs after error correction | 500 | 6.07 | 18.322 | 85.43 | 3,035,485,537 | - | -- |
| PacBio + Hi-C | 143 | 21.2 | 257.6 | 332.794 | 3,035,522,137 | - | 359 |
| PacBio + Hi-C + Optical map | 125 | 63.3 | 242.9 | 330.1 | 3,900,365,415 | - | 421 |
| PacBio + Hi-C + Optical map + five genetic maps | 121 | 25.2 | 260.6 | 334.5 | 3,053,550,485 | 39,262 | 411 |

**Table S2.** Summary of Hi-C sequencing data.

| Libraries | Number of reads | Average read length (bp) |
| --- | --- | --- |
| Pepper library 1 | 451,153,276 | 151 |
| Pepper library 2 | 364,843,160 | 151 |
| Total | 815,996,436 | 151 |

**Table S3.** Summary of optical mapping results.

|  | Molecules |
| --- | --- |
| Total number of molecules | 2,014,828 |
| Total length (Mb) | 476,987.10 |
| Average length (kb) | 236.738 |
| Molecule N50 (kb) | 236.668 |
| Label density (per 100 kb) | 20.878 |
| Coverage of the reference (x) | 157.2 |

**Table S4.** Summary of the final Dempsey genome (v1.0).

| Genomic features | Dempsey genome |
| --- | --- |
| Total length of contigs, Gb | 3.035 |
| Total length of assemblies, Gb | 3.054 |
| Estimated gap size, bp | 18,054,150 |
| Number of contigs | 500 |
| Anchored contigs (%) | 403 (80.6%) |
| Anchored and ordered contigs (%) | 387 (77.4%) |
| Contig N50, Mb | 18.3 |
| Number of scaffolds | 121 |
| Scaffold N50, Mb | 260.6 |
| GC content (%) | 34.7 |
| Repeat elements (%) | 87.84 |
| Number of predicted genes | 39, 262 |

**Table S5.** Details of sequence gaps in the four pepper genomes.

| Chromosome | Dempsey  n (gap size, bp) | CM334 v1.6  n (gap size, bp) | UCD10X v1.0  n (gap size, bp) | Zunla v2.0  n (gap size, bp) |
| --- | --- | --- | --- | --- |
| 1 | 54  (2,273,184) | 29,836  (10,716,924) | 4,197  (7,956,131) | 13,516  (11,953,401) |
| 2 | 25  (516,030) | 17,640  (6,151,211) | 2,597  (4,759,593) | 6,993  (4,873,198) |
| 3 | 40  (29,967) | 30,063  (9,834,082) | 3,933  (7,212,920) | 10,461  (7,584,218) |
| 4 | 19  (1,900) | 18,990  (6,968,221) | 2,837  (5,177,782) | 7,788  (5,613,129) |
| 5 | 47  (551,521) | 19,075  (7,227,703) | 3,044  (5,822,230) | 7,648  (5,573,488) |
| 6 | 27  (2,700) | 22,272  (7,460,941) | 3,166  (5,880,100) | 7,807  (5,224,149) |
| 7 | 23  (1,994,399) | 21,087  (7,706,443) | 3,393  (6,413,927) | 10,899  (11,531,425) |
| 8 | 21  (1,926) | 9,616  (3,511,693) | 2,138  (3,826,456) | 5,662  (3,599,545) |
| 9 | 29  (805,075) | 21,255  (7,867,180) | 3,713  (7,202,877) | 8,304  (5,691,185) |
| 10 | 37  (3,700) | 19,343  (6,962,043) | 3,028  (5,486,551) | 7,343  (5,167,198) |
| 11 | 30  (9,528,121) | 20,735  (7,794,777) | 3,753  (7,737,913) | 10,356  (10,671,361) |
| 12 | 48  (1,355,379) | 22,783  (7,505,152) | 3,066  (5,531,316) | 8,433  (5,882,778) |
| Unanchored | 11  (990,248) | 50,041  (9,535,847) | 13,858  (14,792,092) | 997,588  (71,307,320) |
| Sum | 411  (18,054,150) | 302,736  (99,242,217) | 52,723  (87,799,888) | 1,102,798  (154,672,395) |

**Table S6.** Repetitive DNA contents in 12 pepper accessions.

|  | SINEs  (bp) | LINEs  (bo) | LTR elements  (bp) | DNA transposons  (bp) | Unclassfied (bp) | Total repeats (bp) |
| --- | --- | --- | --- | --- | --- | --- |
| CV3 | 13,923,268 | 94,681,359 | 1,975,128,507 | 273,528,219 | 242,691,267 | 2,599,952,620 |
| Dempsey | 13,797,297 | 93,166,150 | 2,030,500,224 | 280,880,591 | 270,567,640 | 2,688,911,902 |
| I19-702-1 | 15,733,047 | 96,896,302 | 2,040,072,235 | 272,870,332 | 240,400,835 | 2,665,972,751 |
| LaMuyo-01 | 15,291,363 | 97,012,692 | 2,039,611,042 | 271,540,329 | 238,196,361 | 2,661,651,787 |
| Maor | 15,792,957 | 97,598,545 | 2,089,922,467 | 274,440,360 | 258,531,622 | 2,736,285,951 |
| Perennial | 14,472,255 | 93,595,171 | 2,014,078,926 | 274,815,620 | 250,788,209 | 2,647,750,181 |
| PG1 | 15,650,528 | 96,529,013 | 2,065,218,227 | 271,766,958 | 240,613,272 | 2,689,777,998 |
| ThaiHot | 14,100,074 | 97,205,944 | 1,991,224,015 | 281,203,295 | 267,508,998 | 2,651,242,326 |
| UCD10X | 13,437,197 | 94,523,181 | 2,080,498,964 | 288,287,129 | 259,514,855 | 2,736,261,326 |
| CC-090 | 15,364,608 | 96,353,277 | 2,036,408,959 | 277,619,124 | 249,674,565 | 2,675,420,533 |
| CC-260 | 15,743,474 | 96,986,616 | 2,038,767,816 | 278,715,950 | 250,890,293 | 2,681,104,149 |
| MR | 15,426,886 | 95,651,217 | 2,035,469,420 | 277,903,808 | 249,309,808 | 2,673,761,139 |

**Table S7.** Syntenic relationship between pepper genomes and Dempsey.

| Genome | Status | bp | % |
| --- | --- | --- | --- |
| CV3 | Aligned | 2,873,296,034 | 94.09688 |
| I19-702-1 | Aligned | 2,930,102,378 | 95.96 |
| LaMuyo | Aligned | 2,915,596,656 | 95.48 |
| Maor | Aligned | 2,935,550,567 | 96.14 |
| Perennial | Aligned | 2,889,054,841 | 94.61 |
| PG1 | Aligned | 2,923,127,106 | 95.73 |
| ThaiHot | Aligned | 2,842,773,932 | 93.10 |
| UCD10X | Aligned | 2,881,494,192 | 94.37 |
| CC-090 | Aligned | 2,942,437,928 | 96.36 |
| CC-260 | Aligned | 2,934,417,647 | 96.09 |
| MR | Aligned | 2,949,732,200 | 96.60 |
| CA59 | Aligned | 2,928,056,824 | 95.89 |

**Table S8.** Number of SVs in pepper pan-genomes compared with Dempsey genome.

| Number of SVs | | PAV | | CNV | | INV | INDELs | |
| --- | --- | --- | --- | --- | --- | --- | --- | --- |
|  |  | Presence | Absence | Copy  gain | Copy  loss |  | Insertions | Deletions |
| 1 | Perennial | 18,239 | 18,012 | 706 | 765 | 1,795 | 99,107 | 97,158 |
| 2 | ThaiHot | 19,909 | 22,389 | 582 | 1,377 | 744 | 86,313 | 87,005 |
| 3 | CV3 | 15,663 | 18,366 | 439 | 1,868 | 833 | 60,286 | 64,231 |
| 4 | UCD10X | 8,140 | 7,779 | 951 | 2,306 | 7,177 | 31,303 | 41,263 |
| 5 | LaMuyo | 17,124 | 11,913 | 537 | 395 | 1,080 | 22,449 | 23,057 |
| 6 | I19-702-1 | 16,086 | 12,608 | 657 | 442 | 1,335 | 21,746 | 22,775 |
| 7 | PG1 | 19,086 | 10,457 | 513 | 413 | 1,270 | 21,010 | 21,373 |
| 8 | Maor | 17,669 | 12,990 | 503 | 373 | 1,120 | 17,163 | 17,558 |
| 9 | CC-090 | 16,578 | 16,371 | 656 | 673 | 1,370 | 87,985 | 85,996 |
| 10 | CC-260 | 17,456 | 17,340 | 646 | 704 | 1,294 | 94,616 | 91,990 |
| 11 | MR | 15,364 | 15,051 | 638 | 693 | 1,003 | 82,013 | 80,798 |

**Table S9.** Morphological characteristics of pan-genome pepper accessions.

|  | Name | Pungency | Fruit Type | Mature  color | Fruit  position | Clustered  flower |
| --- | --- | --- | --- | --- | --- | --- |
| 1 | Dempsey | Sweet | Blocky | Red | Pendant | Not  fasciculated |
| 2 | CV3 | Hot | - | Red | Erect | Not  fasciculated |
| 3 | ThaiHot | Hot | - | Red | Erect | Fasciculated |
| 4 | Maor | Sweet | Blocky | Red | Pendant | Not  fasciculated |
| 5 | I19-702-1 | Sweet | Blocky | Yellow | Pendant | Not  fasciculated |
| 6 | LaMuyo | Sweet | Blocky | Red | Pendant | Not  fasciculated |
| 7 | PG1 | Sweet | Blocky | Red | Pendant | Not  fasciculated |
| 8 | UCD10X | Hybrid  (Hot × Sweet) | - | Red | F1  (Erect ⅹ Pendant) | Not  fasciculated |
| 9 | Perennial | Hot | - | Red | Erect | Not  fasciculated |
| 10 | CC090 | Hot | - | Red | Pendant | Not  fasciculated |
| 11 | CC260 | Hot | - | Red | Pendant | Not  fasciculated |
| 12 | MR | Hot | - | Red | Erect | Fasciculated |

**Table S10.** PAVs in agronomically important genes.

| Gene | *Pun1* | *BIG GRAIN-like* | *CCS* | *pvr6* | *FA* |
| --- | --- | --- | --- | --- | --- |
| Chr. | 2 | 12 | 6 | 3 | 6 |
| Dempsey | - | - | - | - | - |
| Perennial | 153,498,735 - 153,501,259 (2,525 bp INS) | 221,285,007 - 221,285,578 (572 bp INS) | - | 16,625,597 -16,625,97 (83 bp DEL) | - |
| ThaiHot | 148,781,251 - 148,783,777 (2,527 bp INS) | 212,984,882 - 212,985,461 (580 bp INS) | - | - | 232,833,688 - 232,833,699 (428 bp DEL) |
| CV3 | 149,133,260 - 149,135,786 (2,527 bp INS) | 212,759,471 - 212,760,050 (580 bp INS) | - | 14,505,756 - 14,505,756 (83 bp DEL) | - |
| UCD10X | - | - | - | - | - |
| LaMuyo-01 | - | - | - | - | - |
| I19-702-1 | - | - | 92,306,698 - 92,306,700 (4,430 bp DEL) | - | - |
| PG1 | - | - | - | - | - |
| Maor | - | - | - | - | - |
| MR | 122,409,889 -122,412,415 (2,526 bp INS) | 223,205,523 -223,206,102 (580 bp INS) | - | - | - |
| CC090 | 156,391,012 -156,393,538 (2,527 bp INS) | - | - | - | - |
| CC260 | 153,794,428 -153,796,963 (2,535 bp INS) | - | - | - | - |

-

**Table S11.** Regions with high frequency PAV (top 5% ranked) between hot and sweet peppers.

|  | **Chr** | **Start** | **End** |  | **Chr** | **Start** | **End** |
| --- | --- | --- | --- | --- | --- | --- | --- |
| 1 | 1 | 77200001 | 78400001 | 41 | 9 | 88800001 | 92200001 |
| 2 | 1 | 331000001 | 332200001 | 42 | 9 | 92400001 | 99800001 |
| 3 | 2 | 5600001 | 6600001 | 43 | 9 | 100000001 | 101400001 |
| 4 | 2 | 9600001 | 11000001 | 44 | 9 | 101600001 | 102800001 |
| 5 | 2 | 49000001 | 51000001 | 45 | 9 | 103000001 | 106200001 |
| 6 | 2 | 51200001 | 53200001 | 46 | 9 | 107200001 | 110600001 |
| 7 | 2 | 54200001 | 56000001 | 47 | 9 | 110800001 | 113800001 |
| 8 | 2 | 59800001 | 60800001 | 48 | 9 | 114400001 | 117000001 |
| 9 | 2 | 121800001 | 122800001 | 49 | 9 | 117400001 | 136600001 |
| 10 | 3 | 62200001 | 63200001 | 50 | 9 | 137200001 | 150600001 |
| 11 | 3 | 63600001 | 65000001 | 51 | 9 | 152200001 | 153400001 |
| 12 | 3 | 76400001 | 78400001 | 52 | 10 | 20200001 | 22600001 |
| 13 | 3 | 108800001 | 110400001 | 53 | 10 | 72800001 | 76600001 |
| 14 | 3 | 168200001 | 171000001 | 54 | 10 | 98400001 | 99600001 |
| 15 | 3 | 180800001 | 184400001 | 55 | 10 | 156200001 | 158200001 |
| 16 | 3 | 190200001 | 191200001 | 56 | 11 | 22400001 | 26000001 |
| 17 | 4 | 53600001 | 55000001 | 57 | 11 | 30200001 | 39000001 |
| 18 | 4 | 110200001 | 111200001 | 58 | 11 | 115600001 | 117400001 |
| 19 | 4 | 121800001 | 122800001 | 59 | 11 | 118400001 | 120200001 |
| 20 | 4 | 138400001 | 139600001 | 60 | 11 | 129200001 | 130400001 |
| 21 | 4 | 179800001 | 181400001 | 61 | 11 | 130800001 | 133000001 |
| 22 | 5 | 31200001 | 32600001 | 62 | 11 | 133400001 | 135800001 |
| 23 | 5 | 70400001 | 71400001 | 63 | 11 | 136200001 | 138800001 |
| 24 | 6 | 14400001 | 16200001 | 64 | 11 | 143200001 | 149000001 |
| 25 | 6 | 44600001 | 47200001 | 65 | 11 | 150200001 | 151200001 |
| 26 | 6 | 81200001 | 82400001 | 66 | 11 | 153000001 | 154400001 |
| 27 | 6 | 84600001 | 85600001 | 67 | 11 | 157800001 | 160600001 |
| 28 | 6 | 160200001 | 161200001 | 68 | 11 | 161000001 | 166800001 |
| 29 | 6 | 174800001 | 176800001 | 69 | 11 | 167200001 | 183600001 |
| 30 | 7 | 20600001 | 21800001 | 70 | 11 | 184000001 | 185000001 |
| 31 | 7 | 78400001 | 80800001 | 71 | 11 | 185800001 | 187200001 |
| 32 | 7 | 81400001 | 82400001 | 72 | 11 | 187600001 | 192200001 |
| 33 | 7 | 82800001 | 84400001 | 73 | 11 | 192400001 | 195200001 |
| 34 | 7 | 85000001 | 89000001 | 74 | 11 | 195400001 | 199600001 |
| 35 | 7 | 147600001 | 148600001 | 75 | 11 | 200600001 | 202000001 |
| 36 | 7 | 155800001 | 157400001 | 76 | 11 | 203400001 | 207800001 |
| 37 | 7 | 171200001 | 174400001 | 77 | 11 | 209200001 | 210200001 |
| 38 | 9 | 71800001 | 79800001 | 78 | 11 | 247000001 | 248800001 |
| 39 | 9 | 80000001 | 81600001 | 79 | 12 | 27400001 | 34000001 |
| 40 | 9 | 83000001 | 88200001 |  |  |  |  |

**Table S12.** Previously reported GWAS regions for fruit shape in the Dempsey genome.

| CM334 v.1.6 | | | | Dempsey | | | Reference |
| --- | --- | --- | --- | --- | --- | --- | --- |
| Chr | Start | | End | Chr | Start | End | 42 |
| chr10 | 70,464,537 | | 70,542,670 | 10 | 85,118,399 | 85,195,740 |  |
| chr10 | 157,390,248 | | 157,390,454 | 10 | 113,399,418 | 113,399,213 |  |
| chr11 | 142,383,641 | | 142,466,172 | 11 | 119,496,521 | 119,596,641 |  |
| chr11 | 155,972,621 | | 155,975,491 | 11 | 142,552,992 | 142,555,862 |  |
| chr11 | 162,329,510 | | 162,355,479 | 11 | 151,976,622 | 152,002,590 |  |
| chr11 | 173,493,841 | | 173,510,241 | 11 | 210,539,659 | 210,556,144 |  |
| CM334 v.1.55 | | | | Dempsey | | | 1 |
| Chr | Start | End | | Chr | Start | End |  |
| chr01 | 35,525,837 | 41,894,832 | | 1 | 45,746,747 | 61,357,302 |  |
| chr01 | 156,557,626 | 168,853,068 | | 1 | 190,506,253 | 200,324,119 |  |
| chr01 | 226,143,117 | 264,420,677 | | 1 | 302,031,761 | 309,856,475 |  |
| chr02 | 147,649,687 | 153,821,541 | | 2 | 150,568,278 | 156,993,665 |  |
| chr02 | 157,150,459 | 159,833,718 | | 2 | 160,637,412 | 163,154,977 |  |
| chr03 | 15,589,344 | 19,212,153 | | 3 | 20,848,609 | 25,848,492 |  |
| chr03 | 244,772,375 | 250,606,405 | | 3 | 277,966,820 | 281,301,460 |  |
| chr03 | 252,107,320 | 253,119,732 | | 3 | 285,056,050 | 286,074,223 |  |
| chr04 | 16,968,386 | 58,022,200 | | 4 | 18,642,287 | 46,462,867 |  |
| chr04 | 177,652,216 | 189,238,013 | | 4 | 191,419,142 | 201,863,304 |  |
| chr06 | 81,769 | 4,535,834 | | 6 | 309,922 | 2,412,193 |  |
| chr06 | 219,031,550 | 225,502,847 | | 6 | 230,392,320 | 236,641,421 |  |
| chr06 | 233,198,698 | 235,289,162 | | 6 | 245,311,996 | 248,809,947 |  |
| chr07 | 9,059,522 | 50,078,775 | | 7 | 12,948,314 | 80,733,782 |  |
| chr09 | 4,192,541 | 6,684,873 | | 9 | 7,022,330 | 10,646,564 |  |
| chr11 | 15,134,869 | 39,159,124 | | 11 | 24,579,413 | 50,238,450 |  |
| chr12 | 11,856,333 | 17,964,619 | | 12 | 12,639,836 | 19,755,563 |  |

**Table S13.** Fruit shape related putative candidate genes affected by PAVs in high frequency PAV (top 5% ranked) region between hot and blocky peppers.

| Chr | Start | End | SwissProt Function |
| --- | --- | --- | --- |
| 3 | 63,586,622 | 63,630,653 | RNA-directed DNA polymerase homolog (EC 2.7.7.48) (Reverse transcriptase homolog) |
| 3 | 63,979,749 | 64,003,550 | Uncharacterized mitochondrial protein AtMg00860 (ORF158) |
| 3 | 77,157,516 | 77,170,390 | RNA-directed DNA polymerase homolog (EC 2.7.7.48) (Reverse transcriptase homolog) |
| 3 | 78,046,790 | 78,064,371 | RNA-directed DNA polymerase homolog (EC 2.7.7.48) (Reverse transcriptase homolog) |
| 3 | 109,121,307 | 109,130,271 | RNA-directed DNA polymerase homolog (EC 2.7.7.48) (Reverse transcriptase homolog) |
| 3 | 109,731,599 | 109,745,798 | RNA-directed DNA polymerase homolog (EC 2.7.7.48) (Reverse transcriptase homolog) |
| 3 | 110,356,102 | 110,372,978 | RNA-directed DNA polymerase homolog (EC 2.7.7.48) (Reverse transcriptase homolog) |
| 3 | 168,438,180 | 168,465,131 | RNA-directed DNA polymerase homolog (EC 2.7.7.48) (Reverse transcriptase homolog) |
| 3 | 169,501,423 | 169,503,700 | #N/A |
| 3 | 170,010,774 | 170,022,007 | Clathrin heavy chain 1 |
| 3 | 170,488,205 | 170,492,403 | #N/A |
| 3 | 170,839,962 | 170,845,114 | Retrovirus-related Pol polyprotein from transposon TNT 1-94 [Includes: Protease (EC 3.4.23.-); Reverse transcriptase (EC 2.7.7.49); Endonuclease] |
| 3 | 190,197,398 | 190,201,124 | #N/A |
| 3 | 190,384,266 | 190,387,674 | #N/A |
| 6 | 14,756,699 | 14,771,409 | RNA-directed DNA polymerase homolog (EC 2.7.7.48) (Reverse transcriptase homolog) |
| 6 | 15,558,892 | 15,561,587 | Putative B3 domain-containing protein At5g66980 |
| 6 | 16,006,780 | 16,007,910 | Pentatricopeptide repeat-containing protein At1g62930, chloroplastic |
| 6 | 45,005,324 | 45,007,738 | #N/A |
| 6 | 83,411,270 | 83,424,454 | Delta-1-pyrroline-5-carboxylate synthase (P5CS) [Includes: Glutamate 5-kinase (GK) (EC 2.7.2.11) (Gamma-glutamyl kinase); Gamma-glutamyl phosphate reductase (GPR) (EC 1.2.1.41) (Glutamate-5-semialdehyde dehydrogenase) (Glutamyl-gamma-semialdehyde dehydrogenase)] |
| 6 | 85,379,585 | 85,389,015 | RNA-directed DNA polymerase homolog (EC 2.7.7.48) (Reverse transcriptase homolog) |
| 6 | 175,455,930 | 175,457,618 | #N/A |
| 7 | 21,029,092 | 21,049,126 | #N/A |
| 7 | 79,255,393 | 79,270,364 | RNA-directed DNA polymerase homolog (EC 2.7.7.48) (Reverse transcriptase homolog) |
| 7 | 82,255,291 | 82,271,528 | RNA-directed DNA polymerase homolog (EC 2.7.7.48) (Reverse transcriptase homolog) |
| 7 | 83,167,999 | 83,176,689 | RNA-directed DNA polymerase homolog (EC 2.7.7.48) (Reverse transcriptase homolog) |
| 7 | 83,595,157 | 83,607,548 | RNA-directed DNA polymerase homolog (EC 2.7.7.48) (Reverse transcriptase homolog) |
| 7 | 87,496,830 | 87,532,778 | RNA-directed DNA polymerase homolog (EC 2.7.7.48) (Reverse transcriptase homolog) |
| 7 | 156,563,075 | 156,584,944 | RNA-directed DNA polymerase homolog (EC 2.7.7.48) (Reverse transcriptase homolog) |
| 7 | 172,306,540 | 172,325,288 | RNA-directed DNA polymerase homolog (EC 2.7.7.48) (Reverse transcriptase homolog) |
| 10 | 75,072,522 | 75,091,864 | RNA-directed DNA polymerase homolog (EC 2.7.7.48) (Reverse transcriptase homolog) |
| 10 | 158,028,616 | 158,034,402 | #N/A |
| 11 | 22,971,953 | 22,987,732 | Uncharacterized mitochondrial protein AtMg00750 (ORF119) |
| 11 | 24,828,249 | 24,834,991 | RNA-directed DNA polymerase homolog (EC 2.7.7.48) (Reverse transcriptase homolog) |
| 11 | 36,082,164 | 36,082,978 | #N/A |
| 12 | 27,397,547 | 27,411,054 | Retrovirus-related Pol polyprotein from transposon TNT 1-94 [Includes: Protease (EC 3.4.23); Reverse transcriptase (EC 2.7.7.49); Endonuclease] |
| 12 | 32,007,134 | 32,024,050 | RNA-directed DNA polymerase homolog (EC 2.7.7.48) (Reverse transcriptase homolog) |
